# Subjects treated with expanded non‐genetically modified autologous Natural Killer cells (SNK01) show improved changes in CSF α‐synuclein and in cognitive function

**DOI:** 10.1002/alz.085846

**Published:** 2025-01-09

**Authors:** Clemente Humberto Zúñiga Gil, Blanca Isaura Acosta Gallo, Rufino Menchaca Díaz, Cesar Alejandro Amescua, Sean Hong, Lucia Hui, Hank Lee, Paul Chang, Katia Betito, Paul Y Song

**Affiliations:** ^1^ Tijuana General Hospital, Tijuana, EM Mexico; ^2^ Hospital Angeles, Tijuana, EM Mexico; ^3^ NKGen Biotech, Santa Ana, CA USA

## Abstract

**Background:**

Accumulating evidence suggests that the presynaptic protein α‐synuclein (α‐syn), is involved in the pathophysiology of AD and elevated in the cerebrospinal fluid (CSF). The role of Natural Killer (NK) cells of the innate immune system in AD has largely been overlooked. In a murine model, depletion of NK cells augmented the accumulation of pathological α‐syn. Human NK cells have been shown to efficiently internalize and degrade α‐syn aggregates via the endosomal/lysosomal pathway. This has been proposed as a protective mechanism of NK cells against α‐synucleinopathy by removing harmful α‐syn aggregates from the patient’s brain. Our *in vitro* studies have shown that SNK01, an enhanced autologous non‐genetically modified NK cell product, is able to effectively internalize and degrade α‐syn aggregates.

**Method:**

In this Phase 1 study, SNK01 was administered IV every three weeks for a total of 4 treatments using a 3+3 dose escalation design (1, 2 and 4×10^9^ cells) in subjects with either mild, moderate, or severe AD (Median MMSE of 14). Cognitive assessments and CSF α‐syn analyses were performed at baseline and at 1 and 12 weeks after the final dose. The primary endpoint was safety and secondary endpoints included changes in cognitive assessments and biomarker levels.

**Result:**

Eleven subjects (5 males and 6 females) were enrolled and 10 were evaluable. Median age was 79 years (56 to 85 years). No treatment related adverse events were observed. Despite 70% of subjects being treated at relatively low doses of SNK01, 90% of all evaluable subjects had either stable or improved (±0.1) composite ADCOMS scores at week 11 (one‐week after the final dose), and 60% of subjects (6/10) had a decrease in CSF α‐syn compared to baseline values. In the 5/6 subjects where data was available, these levels continued through week 22 for α‐syn. One additional subject had a decrease in CSF α‐syn compared to baseline values at week 22. At week 11, the decreases in α‐syn corresponded to stable/decrease in ADCOMS in 5/6 subjects.

**Conclusion:**

SNK01 was safe and well tolerated. SNK01 appears to have clinical activity in AD while also reducing α‐syn protein levels in the CSF.

